# Antiepileptic Drugs for De Novo Seizure Prevention After Craniotomy: A Systematic Review and Network Meta-Analysis of Current Evidence

**DOI:** 10.3390/jcm14217854

**Published:** 2025-11-05

**Authors:** Georgia Tsaousi, Adriani Nikolakopoulou, Parmenion P. Tsitsopoulos, Chryssa Pourzitaki, Dimitrios Mavridis, Anna Bettina Haidich

**Affiliations:** 1 Department of Anesthesiology and ICU, School of Medicine, Aristotle University of Thessaloniki, 54124 Thessaloniki, Greece; 2 Laboratory of Hygiene, Social & Preventive Medicine and Medical Statistics, School of Medicine, Aristotle University of Thessaloniki, 54124 Thessaloniki, Greece; anikolakopoulou@auth.gr (A.N.); haidich@auth.gr (A.B.H.); 3 Second Department of Neurosurgery, School of Medicine, Aristotle University of Thessaloniki, 54124 Thessaloniki, Greece; ptsitsopoulos@auth.gr; 4 Laboratory of Clinical Pharmacology, School of Medicine, Aristotle University of Thessaloniki, 54124 Thessaloniki, Greece; chpour@auth.gr; 5 Department of Primary Education, University of Ioannina, 45110 Ioannina, Greece; dmavridi@uoi.gr

**Keywords:** anticonvulsant, antiepileptic, seizure, prophylactic, brain tumor, craniotomy

## Abstract

**Objective**: We aimed to systematically evaluate the available clinical evidence concerning the comparable efficacy and safety of currently used anti-epileptic drugs (AEDs) for seizure prophylaxis in patients undergoing craniotomy for brain tumor excision and synthesize this with a network meta-analysis (NMA). **Methods**: A systematic literature review was performed to identify randomized controlled trials (RCTs) relevant to the prophylactic use of AEDs in seizure-naïve patients subjected to brain tumor excision. Total, early, or late post-craniotomy seizures constituted primary outcome measures, while mortality and treatment-related adverse effects served as secondary endpoints. Pairwise and network meta-analysis were conducted for each pair of interventions to obtain ‘direct’ treatment effect estimates, while NMA was employed to assess the relative efficacy and safety of prophylactic use of AEDs in post-craniotomy epilepsy management in brain tumor cases. **Results**: Twelve eligible RCTs involving 10 interventions were retrieved. Levetiracetam (OR 0.08; 95%CI 0.02–0.43) and phenytoin (OR 0.43; 95%CI 0.20–0.91) showed superior efficacy over placebo on early seizure control, while none of the applied interventions demonstrated any significant effect on late seizures versus placebo. With the single exception of carbamazepine (OR 3.29; 95%CI 1.21–8.91), none of the implemented AEDs exerted a notable effect on mortality. Phenytoin presented a higher incidence of treatment-related AEs, imposing drug discontinuation compared to other treatment regimens, yet this effect did not reach statistical significance. **Conclusions**: Our NMA indicates that, in seizure-naive individuals subjected to brain tumor excision, levetiracetam and phenytoin effectively prevent postoperative short-term seizure activity. Notwithstanding the fact that levetiracetam presents an enhanced safety profile over other AEDs, no statistical superiority could be demonstrated. PROSPERO registration CRD42022377136.

## 1. Introduction

Epileptic seizures constitute a well-recognized clinical entity in patients harboring a brain tumor [1,2,3]. An epileptic seizure could be a common clinical manifestation at the onset of tumor progression (30–80%), while a minority of patients (10–30%) with brain tumors develop late seizures [4,5]. Notably, the mass lesion per se, in conjunction with its surgical resection, could predispose to the aggravation of post-craniotomy seizure activity [5]. Although there is no clear cutoff point for the distinction between early and late seizures postoperatively, conventionally, the appearance of seizures within 1 week after surgery is defined as early seizures, while those occurring later are defined as late seizures [6].

The consequences of persistent seizure activity early after craniotomy involve the development of brain edema, traumatic brain damage, and aspiration, as well as an adverse impact on the neurological recovery and long-term quality of life of the affected individuals [6]. The goal of preventive anti-epileptic drugs (AEDs) is to reduce the development of early seizures while minimizing the incidence of late-onset seizures without the occurrence of profound adverse effects [7]. Nonetheless, it should be emphasized that only limited data affirm the efficacy of this practice, based on the absence of a class I study addressing this question [8]. On the other hand, each AED incurs potential harm, which should be taken into account before the commencement of antiseizure prophylaxis. Given the hazards of AED side effects, even if AEDs are given preoperatively in seizure-free patients with brain tumors, it is recommended that they be discontinued during the first week after surgery [9].

Nonetheless, AEDs are ordinarily commenced perioperatively, with their use being maintained for at least 1 year, provided that no seizure recurrence is recorded during that period [10]. In the current literature, several systematic reviews and meta-analyses have attempted to investigate the efficacy and safety of pairwise monotherapy comparisons or the direct comparisons of anticonvulsants to a placebo using individual participant data; however, their findings are inconclusive [11,12,13,14,15,16]. In the absence of robust evidence in this area, current practice relies on guidelines based on limited and low-quality clinical trials [5,7,17,18,19,20].

Furthermore, direct evidence from randomized controlled trials is not available for some drug comparisons or comparisons to a placebo, which might be of great importance in order to expand our knowledge in this area [11,21,22,23]. Thus, this study aims to systematically evaluate the available clinical evidence concerning the comparative efficacy and safety of all anticonvulsants currently used as prophylaxis for early or late de novo seizure occurrence compared with a placebo in patients undergoing elective craniotomy for brain tumor excision and synthesize this with a network meta-analysis.

## 2. Materials and Methods

### 2.1. Search Strategy

This review was performed following the Cochrane Handbook for Systematic Reviews of Interventions [24]. Furthermore, the Preferred Reporting Items for Systematic Reviews and Meta-Analyses (PRISMA) standards [25] were followed (Table S1: PRISMA Checklist in Supplementary Materials), as were the subsequent additions for Network Meta-Analyses (NMA) [26]. The protocol of this review has been registered in the International Prospective Register of Systematic Reviews (PROSPERO) with the identifier CRD42022377136.

A methodological and comprehensive search approach was devised to retrieve all relevant papers that met the study’s objectives. A meticulous database search involving the National Library of Medicine’s PubMed, Scopus, Cochrane Central Register of Controlled Trials (CENTRAL), and Web of Science from their inception to 15 July 2025 was conducted to detect relevant papers. Free-text and medical subject heading (MeSH) terms such as “craniotomy”, “brain surgery”, “brain tumor”, “epilepsy”, “seizure”, “antiepileptic”, “anticonvulsant”, “prophylactic”, “prophylaxis”, “adverse effects” or “side effects” using appropriate Boolean operators, were applied to the literature search strategy to identify articles relevant to the objectives of this review (Tables S2, S3 and Figure S1 in Supplementary File). Our search strategy also incorporated several trial registries, namely Clinicaltrial.gov, the EU Clinical Trials Register, and the International Clinical Trials Registry Platform, to track any unpublished eligible trials. The last electronic search was performed on 10 August 2025.

### 2.2. Criteria for Considering Studies for This Review

Successfully screened studies were subsequently assessed for inclusion if they conformed to the following criteria: (1) original double-blind, single-blind, or unblinded RCTs involving a parallel design comparing at least two of the currently licensed AEDs prescribed as monotherapy for perioperative seizure prophylaxis in patients subjected to supratentorial or infratentorial craniotomy; (2) adult patients (age ≥ 18 years) undergoing brain tumor surgery with a portion of at least ≥50% of them being seizure-naïve; (3) direct comparison of an active treatment arm (any AED) with the control arm (alternative AED type, or no prophylaxis involving placebo and no-treatment); (4) provision of complete data on at least one of the predefined primary outcome measures; and (5) full-text publication with no language restriction. Articles focusing on the treatment of status epilepticus elicited by brain injury or involving a history of seizure disorders were excluded.

### 2.3. Outcome Measures

The primary study outcomes involved the presence of total and early or late seizure activity, defined as seizures occurring within the first postoperative week or beyond this period, respectively. The efficacy of the implemented AED was assessed by seizure freedom or a decline in seizure activity of ≥50% of the cases (seizure response).

As supplementary relevant secondary endpoints served: (1) mortality during follow-up assessment, and (2) the occurrence of major adverse effects related to the use of AEDs leading to drug discontinuation. Concerning safety outcomes, we also registered the proportion of individuals experiencing at least one major adverse event necessitating treatment or discontinuation of the implemented AEDs.

### 2.4. Screening and Selection Process

Complying with the selection strategy, two investigators (G.T. and C.P.) independently screened the titles and abstracts of all retrieved publications to detect articles potentially relevant to AED prophylaxis in patients subjected to brain tumor excision. Duplicate manuscripts and articles with clearly unrelated content were promptly deleted. Multiple records from the same research were compiled at the end of the data collection process. If eligibility could not be ascertained from the article title or abstract, the entire document was acquired, and studies were evaluated for eligibility based on their adherence to the stated inclusion criteria and overall clinical significance. Reference lists of recovered articles were hand-searched to ensure no relevant publications were overlooked. Disagreements in the screening and selection process were resolved by discussion or were arbitrated by the senior reviewer (D.M.) until consensus was reached.

### 2.5. Data Extraction

To record all pertinent data, a custom-designed abstraction form was employed. Data of interest included publication details (author, year of publication, study design, number of participants), brain pathology, details on research and control arms (type, dose/frequency, time of treatment commencement, and duration of AED prophylaxis), duration of follow-up, and findings relevant to the primary or secondary endpoints.

### 2.6. Quality Assessment

The Cochrane Collaboration Risk of Bias tool Version 2 for randomized trials (RoB2) [27] was applied to critically appraise the quality of the incorporated RCTs concerning the following domains: randomization process, timing of identification or recruitment of participants, deviations from intended interventions, missing outcome data, measurement of the outcome, and selection of the reported result. Each item was assigned a low, some concerns, or high risk of bias. If a study met all of the low-risk criteria, it was classified as low risk; if it met any high-risk domains, it was classified as high risk. The risk of bias was assessed as having some concerns in all other circumstances. RoB2 judgments targeted the effect of adherence (per-protocol) assessment and were outcome-specific. The quality of evidence was tabulated using the Confidence in Network Meta-Analysis (CINeMA) software (http://cinema.ispm.unibe.ch, CINeMA 2.0.0, accessed on 22 May 2025) [28]. An OR lower than 0.7 (or higher than 1.43) across primary outcome endpoints was judged clinically significant.

In a further attempt to reduce the influence of subjective evaluation, the methodological quality attributed to each trial was judged separately by three reviewers (G.T., A.N., C.P.), while any disagreements that arose following the evidence evaluations were handled by consensus.

### 2.7. Statistical Synthesis

We conducted a network meta-analysis for each outcome using R software (version 4.5.1, http://www.r-project.org, accessed on 17 August 2025) using “meta” and “netmeta” packages.

Initially, a pairwise meta-analysis was conducted for each pair of interventions using a random-effects model to yield odds ratios (ORs) with 95% confidence intervals (CIs) of the relative treatment effects. The restricted maximum likelihood (REML) method was used to estimate heterogeneity for each pairwise comparison [29]. We then conducted a network meta-analysis using the REML method in sensitivity analyses in addition to the DerSimonian–Laird estimator (Tau^2^) for heterogeneity assessment across all comparisons, though this estimator can underestimate Tau^2^ in small and sparse networks. The node-splitting approach was used in each closed loop to assess the disparities between direct and indirect evidence [30].

The ranking probability for all AEDs of being at each possible rank for each intervention was also assessed. The relative plots (rankograms) and *p-scores* were utilized to create a hierarchy of competing interventions, with a higher ranking representing superior efficacy [31].

A funnel plot was constructed to detect small-study effects, and tests for funnel plot asymmetry determination were planned if at least ten studies were included in the meta-analysis. In an attempt to disentangle publication bias from small-study effects, contour-enhanced funnel plots were created.

## 3. Results

### 3.1. Search Process

**Figure A1:**
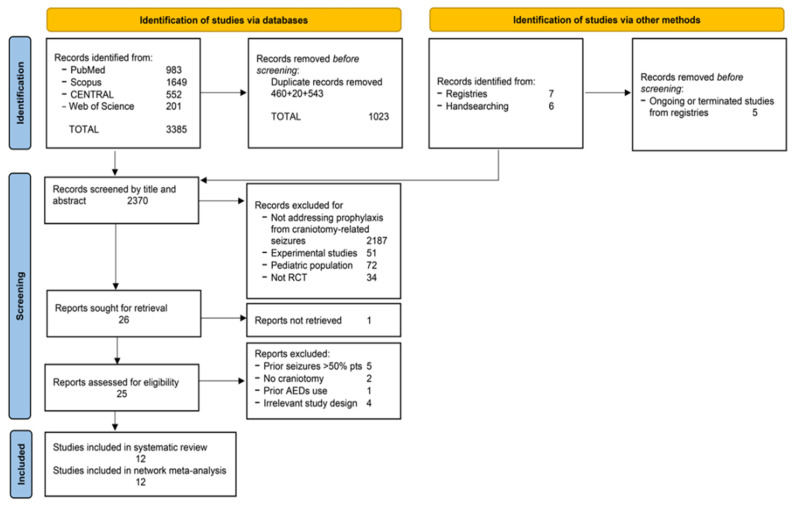
PRISMA flow chart of the study selection process.

The primary database search returned a total of 4976 citations. Thirteen additional publications were detected from registries or hand-searching through reference lists of the included trials. Among them, 5 were promptly discarded for involving early-terminated or ongoing studies. After removing all duplicates and screening titles/abstracts, the full-text versions of the remaining 26 publications were examined for possible eligibility. A single paper was excluded from consideration on the basis that a full-text version could not be acquired, leaving 25 papers for further assessment. At this point, 13 full-text articles were discarded due to methodological constraints, namely the inclusion of patients with pre-existing seizure activity in >50% of patients, prior use of anticonvulsant medication, no craniotomy procedures, irrelevant study design, or non-full-text publications. Thus, 12 RCTs examining the effectiveness of AEDs on post-craniotomy seizures (PCS) involving a total of 2011 participants were selected for inclusion in the final qualitative appraisal [32,33,34,35,36,37,38,39,40,41,42,43]. A flowchart describing the literature search process is presented in Figure A1.

### 3.2. Description of Included Trials

All included studies but two [34,42] employed a two-arm study design. Phenytoin constituted the main anticonvulsant medication being used, while the tested arms involved an active comparator [32,33,34,35,36,37,38], a no-prophylaxis control group [34,39,40,41], or the combination of an active comparator and a no-prophylaxis control group [34,42]. Valproate [32,33], carbamazepine [34], levetiracetam [35,36,37], gabapentin [38], and phenobarbital [42,43] served as active controls. A two-arm study assessed the comparable use of zonisamide to phenobarbital [43]. It should be pointed out that a single study [42] reported a pooled analysis of the efficacy and safety of either phenytoin or phenobarbital.

The duration of anti-seizure treatment varied considerably among studies, ranging from 3 days up to 24 months, while in one trial, the treatment period was not clearly identified [42]. Regarding the follow-up determination, early seizures were consistently defined as seizures occurring within 3 to 7 days post-surgery, while late seizure assessment demonstrated a considerable variation among the included studies, ranging from 1 month to 4 years. Early seizures were evaluated as a primary point of interest in 11 studies [32,33,35,36,37,38,39,40,41,42,43], while complete data on both primary outcome parameters were provided by 7 trials [32,37,38,39,40,41,42,43]. The occurrence of late seizures constituted the single endpoint of the efficacy of seizure prophylaxis in only a three-arm RCT [34]. Two RCTs used either tolerability and safety of the administered AEDs [35] or postoperative pain control [38] as the primary study endpoints, while seizure control served as a secondary study outcome.

It should be noted that no uniformity in drug administration route and dosing regimen could be detected among the selected RCTs. In more than 50% of the studies, anti-seizure prophylaxis was implemented perioperatively [33,34,35,37,41,42,43]. In three RCTs, anticonvulsants were administered only postoperatively [32,39,40], in one, only preoperatively [38], while in the remaining one, the tested drugs were commenced intraoperatively and continued thereafter [36].

The study group consisted of seizure-naïve participants in nine trials [32,33,34,37,38,39,40,41,43]. Although Franceschetti et al. [42] included both seizure-naïve patients as well as those with preoperative seizures, they analyzed separately the seizure-naïve group compared to the control one in order to conform to our inclusion criteria. Similarly, Iuchi et al. [36] included people who had preoperative seizures, but the trial authors provided the results of subgroup analysis for participants with no preoperative seizures. Finally, a single trial [35] also incorporated patients with seizure activity before randomization, but the percentage in both treatment arms was considerably low (5% in each group). Study characteristics and individual study outcomes are detailed in Appendix A (Table A1 and Table A2).

### 3.3. Network Plots

**Figure 1:**
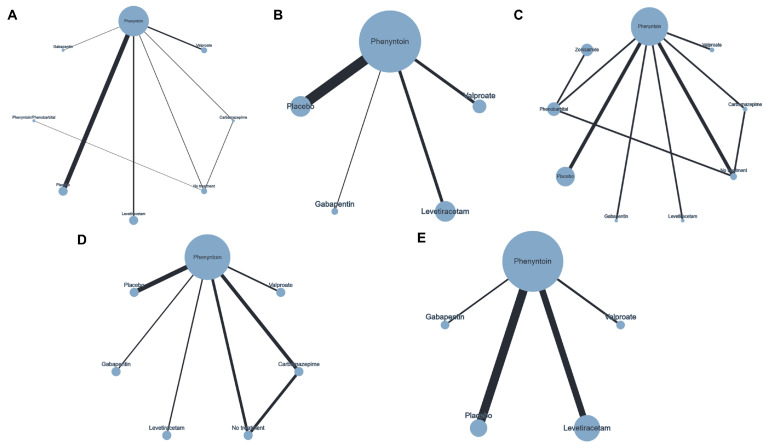
Network plot of the efficacy and safety of the assessed anti-epileptic drugs (AEDs). Total (**A**), early (**B**), and late (**C**) seizure control as well as mortality (**D**), (**E**), and adverse events imposing the discontinuation of AEDs. The width of each edge is proportional to the number of randomized controlled trials comparing each pair of treatments, and the size of each treatment node is proportional to the number of randomized participants (sample size).

The network plots showing the evidence for assessing the impact of anti-seizure prophylaxis on primary and secondary outcomes are presented in Figure 1. No closed loop between treatment arms existed in early seizure control and major adverse effects plots.

### 3.4. Primary Outcomes

#### 3.4.1. Total Seizures

**Figure 2:**
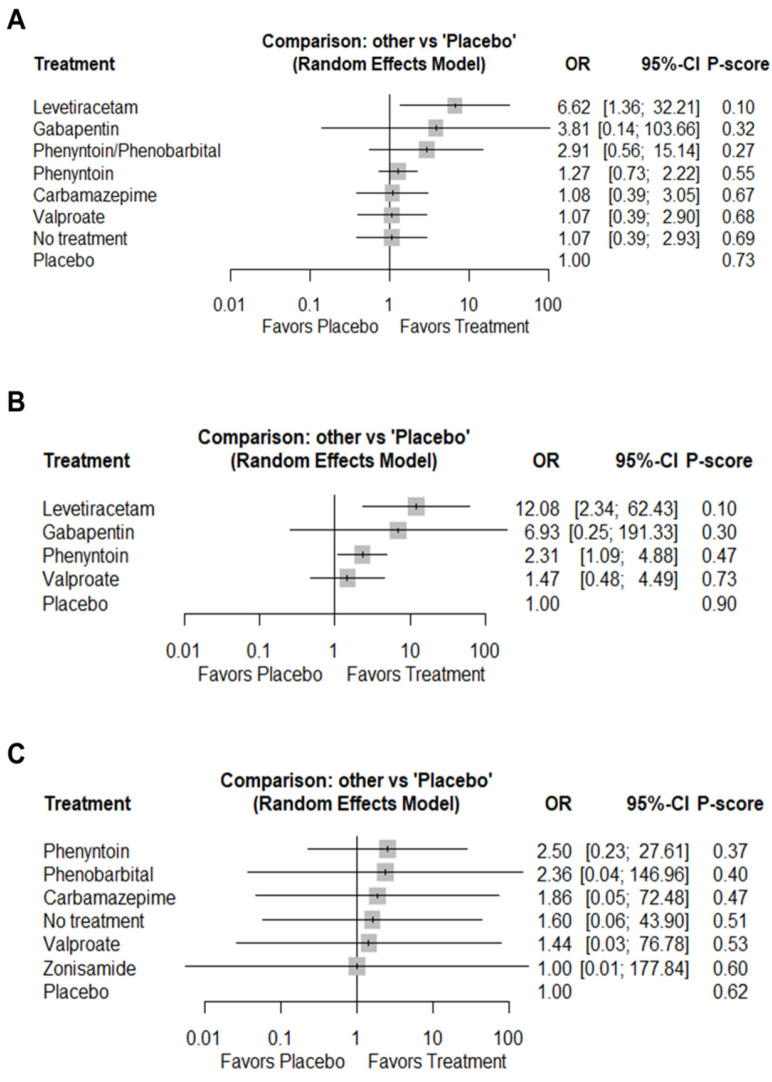
Forest plot of each treatment modality versus placebo for total (**A**), early (**B**) and late (**C**) seizure control.

**Figure A2:**
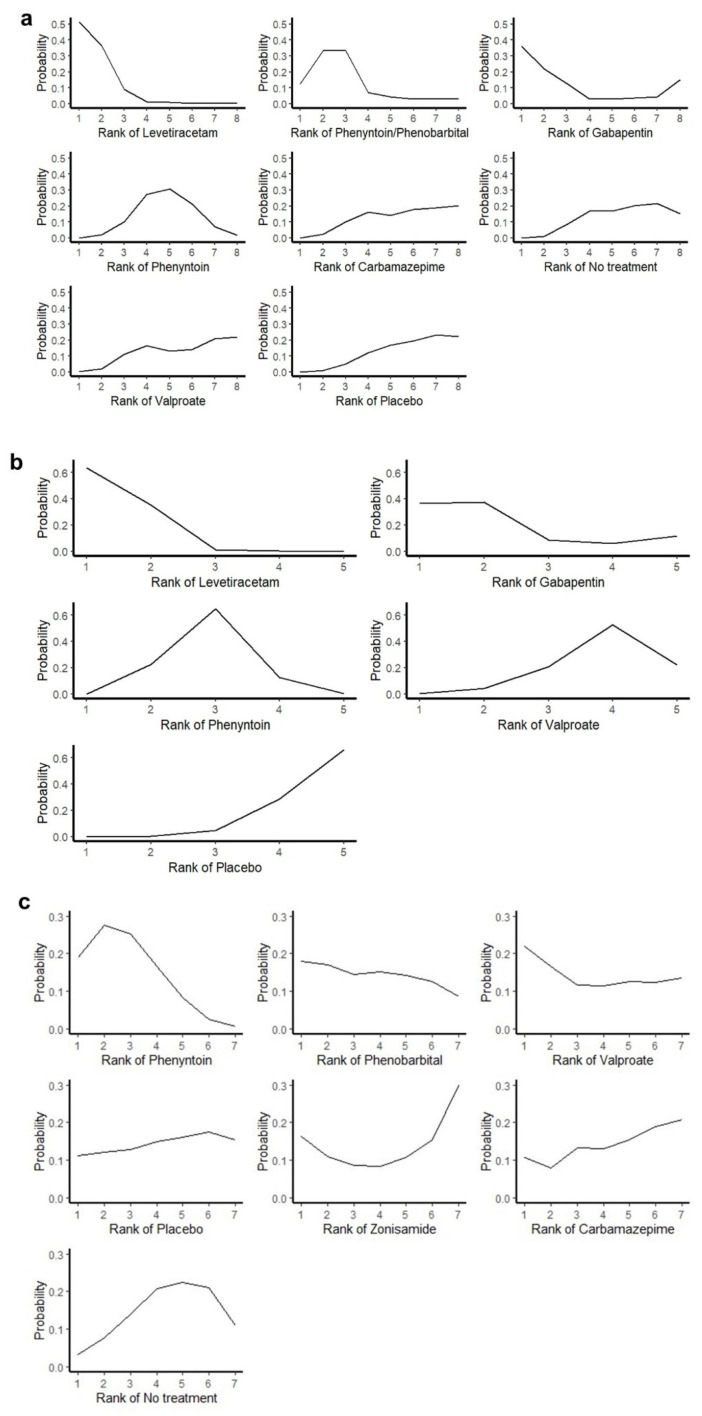
Rankograms illustrating the relative ranking of each treatment modality for the control of (**a**) total, (**b**) early, and (**c**) late seizures.

**Figure A3:**
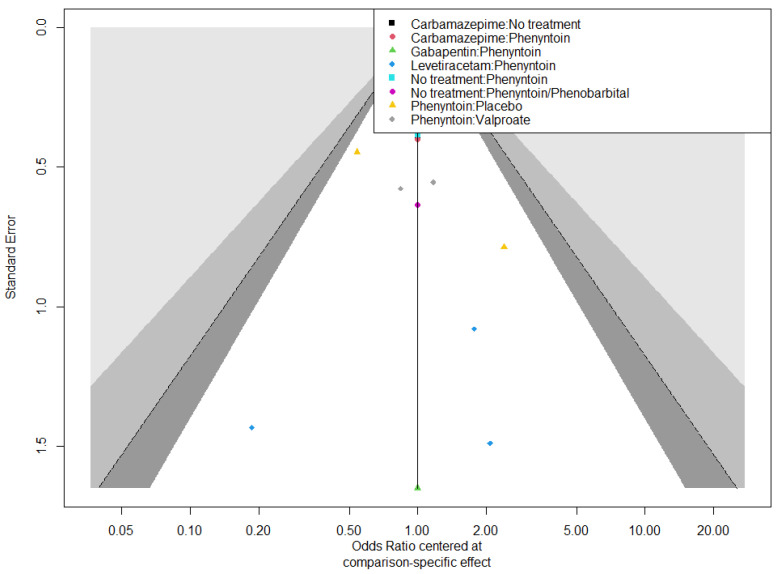
Contour-enhanced funnel plot for total seizures. Different shades indicate *p*-value ranges (white for >0.10, gray for 0.05–0.10, dark gray for 0.01–0.05, and black for <0.01).

Eleven RCTs comprising 13 pairwise comparisons were assessed in the meta-analysis model. Only levetiracetam (OR 6.62; 95%CI 1.36–32.21) demonstrated a notable superiority compared with placebo in terms of total PTS occurrence and was ranked as the most effective intervention (Figure 2A and Figure A2a). In line with previous findings, NMA showed that levetiracetam was more likely to reduce total PCS compared with phenytoin [OR 5.21; 95%CI 1.19–22.91], valproate [OR 6.19; 95%CI 1.13–33.77], no treatment [OR 6.20; 95%CI 1.13–34.09], as well as placebo [OR 6.62; 95%CI 1.36–32.21]. No other significant differences were found; yet, estimates were very imprecise (Table 1). No particular heterogeneity (τ^2^ = 0.038; I^2^ = 9.1%) or asymmetry due to publication bias (Bias estimate = 0.056; SE = 0.462; *p* = 0.905) was encountered in the NMA model (Figure A3).

#### 3.4.2. Early Seizures

All included RCTs but one [34] assessed the impact of anticonvulsant prophylaxis on early postoperative seizure occurrence. Although the early-seizure network contained no closed loops, we conducted an NMA anchored on common comparators (τ^2^ = 0); however, local inconsistency could not be assessed. Levetiracetam (OR 12.08; 95%CI 2.34–62.43) and phenytoin (OR 2.31; 95% CI 1.09–4.88) showed superior efficacy over placebo on early seizure control, while the favorable effect registered for gabapentin and valproate was not statistically significant (Figure A2b). Although local inconsistency could not be assessed, gabapentin was ranked as a second choice after levetiracetam, yet the probability was quite low (<0.4) (Figure A2b). According to the league table (Table 2) levetiracetam was superior to phenytoin [OR 5.23; 95%CI 1.21–22.57], valproate [OR 8.23; 95%CI 1.53–44.29], or placebo [OR 12.08; 95%CI 2.34–62.43] for early seizure control, while phenytoin presented higher efficacy only compared to placebo (OR 2.31; 95%CI 1.09–4.88). Model heterogeneity was negligible (τ^2^ = 0; I^2^ = 0%). Because the network plot did not form a closed loop, inconsistency was not evaluated.

#### 3.4.3. Late Seizures

**Figure A4:**
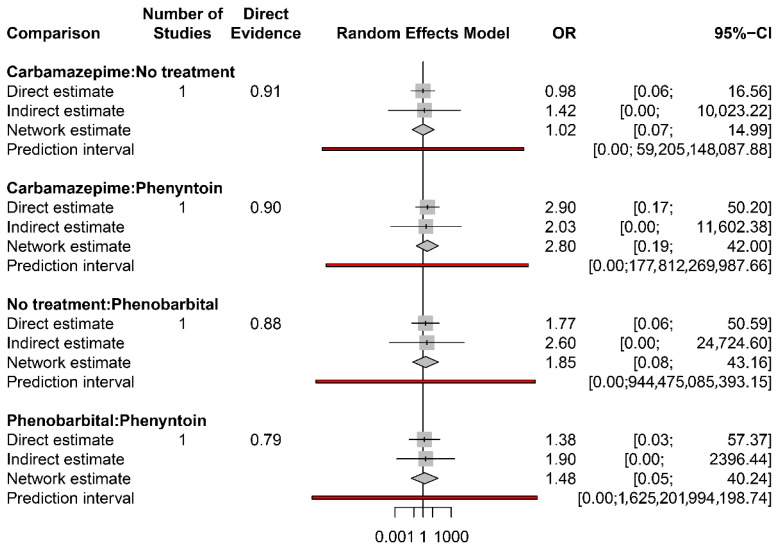
Forest plot for direct and indirect estimates for each pair of comparisons for late seizures, using separate indirect from direct evidence (SIDE) and back-calculation methods. Red lines represent prediction intervals.

Importantly, no difference in late seizure occurrence between treatment arms was reported [32,34,35,37,38,40,41,42,43], a finding further confirmed by the synthesis of relevant data from 8 RCTs and 10 pairwise comparisons, which showed that none of the tested treatment regimens demonstrated any notable superiority in terms of late PCS occurrence (Figure A2c). Similarly, when we assessed the relative effectiveness of each pair of comparisons, we failed to detect any notable difference between the tested AEDs in late seizure control (Table 3). Between-study heterogeneity was substantial (τ^2^ = 2.04; I^2^ = 67.3%). The direct/indirect effect for both loops (phenytoin, carbamazepine, no treatment, and phenytoin, no treatment, phenobarbital) did not show any evidence of inconsistency (χ^2^ statistic = 0.003; *p* = 0.961) (Figure A4). Of importance, in the majority of comparisons, data were provided by a single trial, a small number of participants, or both.

Finally, a single study [43] comparing zonisamide and phenobarbital failed to detect any notable effect regarding total, early, and late PCS control.

### 3.5. Secondary Outcomes

#### 3.5.1. Mortality

**Figure A5:**
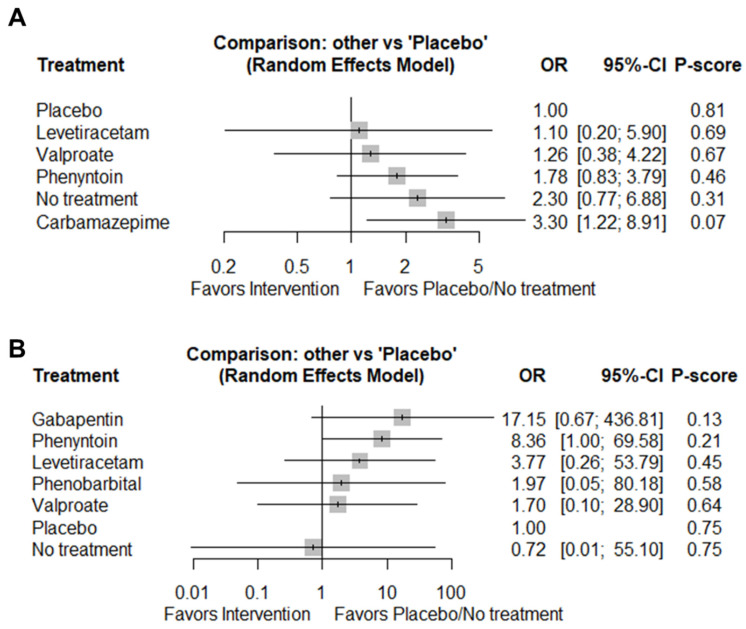
Forest plot of each treatment modality versus placebo for mortality (**A**) and major adverse events leading to drug discontinuation (**B**).

**Figure A6:**
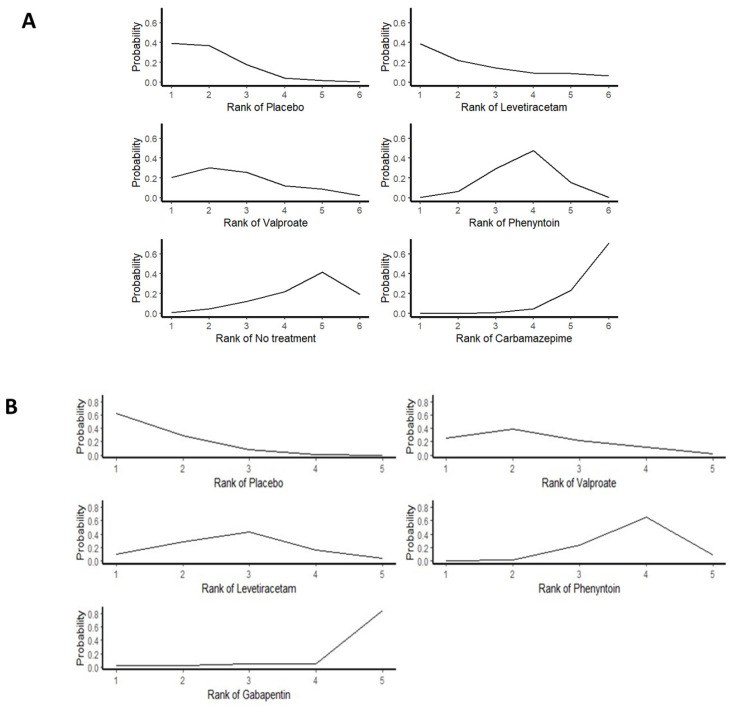
Rankograms demonstrating the relative ranking of each treatment modality regarding the mortality (**A**), and major adverse effects imposing drug discontinuation (**B**).

From the limited number of studies (4 RCTs, 6 pairwise comparisons) [32,34,35,40], carbamazepine presented a higher tendency compared to the other tested AEDs for mortality aggravation (OR 3.30; 95%CI 1.22–8.91). Surprisingly, the no-treatment arm presented a two-fold higher odds ratio than placebo for an adverse effect on mortality (Figure A5A). The ranking of the tested interventions identified placebo as the intervention with the most favorable effect on mortality (Figure A6A). Nonetheless, the league table revealed that none of the tested AEDs exerted any notable effect compared to placebo or to other active drugs (Table A3), while the heterogeneity/inconsistency of this model could not be estimated.

#### 3.5.2. Major Adverse Effects

All included studies but one [39] assessed the impact of anticonvulsant treatment on adverse effects. The most common adverse effects related to phenytoin implementation were liver dysfunction, skin rashes, allergic reactions, postoperative nausea and vomiting, or drug intoxication [32,34,35,36,40,41]. The use of valproate elicited thrombocytopenia or liver dysfunction [32], while levetiracetam induced limited adverse effects involving mainly delirium, headache, and mood disturbances [35].

Major adverse effects necessitating the discontinuation of the applied anticonvulsant were reported in 7 trials [32,35,36,37,38,40,41], while one study reported the development of adverse effects in the whole study population [34]. The incidence of major adverse effects ranged from 0 to 10%, while up to 47% of the patients treated by AEDs suffered from minimal adverse events. Of importance, none of the applied AEDs exerted a statistically significant unfavorable effect on the occurrence of serious adverse effects, imposing the discontinuation of AEDs (Figure A5B). As anticipated, placebo (p-score = 0.880) ranked as the safest practice concerning the occurrence of major adverse effects leading to drug discontinuation, yet the local inconsistency could not be assessed in the model (Figure A6B).

Using the league table (Table A4) to demonstrate the relative contribution of each pair of comparisons, we failed to detect any notable effect of the tested AEDs on major adverse effects, with the model estimates being considerably imprecise. Because the network plot did not form a closed loop, inconsistency was not evaluated.

### 3.6. Risk of Bias Assessment and Confidence in Evidence

**Figure 3:**
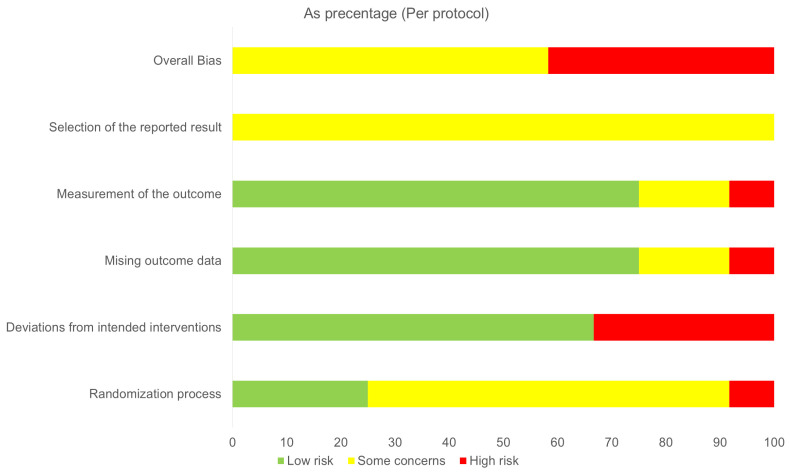
Risk of bias assessment of the included studies according to the RoB-2 Cochrane Bias Tool.

**Figure A7:**
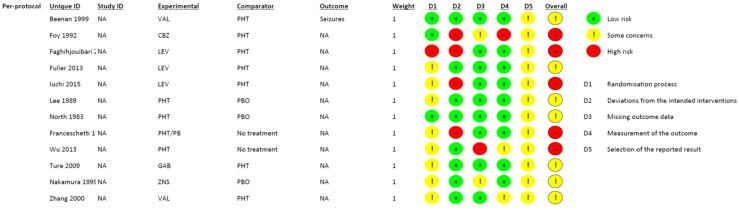
A traffic-light matrix plot of risk of bias assessment for each study according to the RoB-2 Cochrane Bias Tool.

The estimation of the overall risk bias being assessed by the RoB 2 tool revealed that seven out of 12 RCTs (58%) were rated as having “some concerns”, while the remaining ones were classified as “high risk” of bias (Figure 3 and Figure A7). Approximately 35% of the studies were graded with a high risk of bias in the component of adherence to study protocol, while all included studies failed to provide clear data regarding the selectivity of the reported outcomes.

The final estimation of the confidence rating regarding primary outcomes with CINeMA revealed that the relevant models were predominantly of moderate to low quality, with main concerns involving within-study bias and incoherence. Evidence of imprecision was noted, likely due to the limited number of trials available for comparison (Table A5). The comparisons relevant to secondary outcomes were graded with low to very low confidence ratings, with within-study bias, imprecision, and incoherence being the domains presenting the major quality issues (Table A6). Of note, incoherence in both primary and secondary outcomes could not be evaluated due to the failure of closed-loops formation.

## 4. Discussion

This network meta-analysis showed that levetiracetam constituted the only AED demonstrating obvious superiority in total and early seizure control after brain tumor excision over no prophylaxis or other established AEDs. However, phenytoin exerted a positive effect on early seizure activity compared only with the no prophylaxis group. None of the commonly applied AEDs presented any notable advantage in terms of post-operative seizure occurrence, mortality, or adverse effects compared to placebo. Of note, levetiracetam ranked as the top choice for total and early seizure control as well as mortality reduction compared to other anticonvulsant regimens.

Experts in neuroscience have been intensively investigating the safety and effectiveness of different AEDs in preventing post-craniotomy epilepsy [14,19,44,45]. Considering that epilepsy might develop later in the course after craniotomy surgery, prophylactic AEDs have been increasingly prescribed for newly diagnosed primary or metastatic brain tumors, even in the absence of a seizure, to reduce the risk of developing seizure activity following craniotomy surgery. However, this practice remains controversial, as shown by relevant meta-analyses [5,12,14,23,45,46,47]. A plausible explanation for the failure of earlier studies to demonstrate the efficacy of prophylactic AEDs is that the investigators assessed the use of phenytoin, a traditional AED, for craniotomy-related epilepsy prevention rather than more recent AEDs, such as levetiracetam [47].

Taking into account the inconclusive existing evidence, the current practice seems to be determined by the recommendations of the Society for Neuro-Oncology (SNO) and the European Association of Neuro-Oncology (EANO) [48], and the American Association of Neurological Surgeons/Congress of Neurological Surgeons (AANS/CNS) [49], as well as the clinical experience or subjective judgments of the doctor. Preventive administration of AEDs is still often practiced by neurosurgeons—even in the absence of Class I evidence—a practice guided by tumor size, histology, and location as well as the extent of peritumoral edema [3,4,14].

A relevant survey conducted in 2005 demonstrated that approximately 70% of neurosurgeons prescribed prophylactic AEDs; a practice more frequently applied for intra-axial tumors (70%) and less frequently for stereotactic biopsies (21.4%) [50]. A decade later, Dewan et al. [49] showed that levetiracetam is the medicine of choice for more than 63% of neurosurgeons who treat seizure-naïve patients being administered from one week to six weeks following tumor excision. Nonetheless, a recently conducted UK survey [8] reported that seizure prophylaxis is less commonly applied by neurosurgeons, with 59% and 79% of them not prescribing prophylactic AEDs for glioma and meningioma resections, respectively. In particular, the implementation of seizure prophylaxis in patients undergoing craniotomy who have not yet experienced a seizure could not be substantiated as routine practice by current research, based on the claim that the postoperative seizure rate is not high enough to compensate for any potential negative effects of AEDs [5].

Our NMA confirmed current evidence on this topic, as the prophylactic use of the commonly used AEDs in seizure-naïve patients failed to exert any beneficial effect on post-craniotomy epilepsy occurrence. The single exception was levetiracetam, which exhibited a statistically significant reduction in both early and total seizures compared not only to no prophylaxis but also to phenytoin or valproate and emerged as a potential first-line monotherapy according to its efficacy ranking. Phenytoin was shown to be beneficial for early seizure control only compared with no prophylaxis. Nonetheless, the impact of levetiracetam on late seizure occurrence could not be ascertained. It should be underlined that the early seizures model demonstrated an enhanced quality in the analysis of late seizure control.

The beneficial effect of levetiracetam on early seizure activity has important clinical implications, considering that the highest risk of postoperative seizures occurs within the first week of surgery [12,51]. However, two recently published meta-analyses on the prophylactic role of levetiracetam in post-craniotomy epilepsy in seizure-naïve patients provided conflicting evidence. In detail, Wang et al. [15] failed to demonstrate the efficacy of levetiracetam for early seizure prophylaxis in seizure-naïve glioma patients, while Lee et al. [47] showed that levetiracetam yielded a superior impact on seizure prevention for nontraumatic pathology compared to phenytoin with a concomitant reduction in serious adverse effects that led to drug discontinuation. It should be noted that both of these meta-analyses incorporated a limited number of RCTs; thus, the majority of evidence was extracted from observational retrospective studies.

Another important finding of our NMA was that phenytoin exhibited a positive effect only on early seizure activity over no prophylaxis. Although this anticonvulsant drug has long been considered the prototype AED for mitigating seizure activity, it failed to demonstrate any profound superiority in terms of efficacy over other tested AEDs or no-prophylaxis in our clinical setting.

Phenytoin has historically been preferred in neurosurgery, as it presents a well-established therapeutic serum concentration range, does not impair the level of consciousness, and can be monitored easily [14,52]. Nonetheless, phenytoin presents numerous drawbacks, including its unpredictable nonlinear pharmacokinetics, risk of adverse reactions, drug interactions, and reported negative influence on outcome in stroke and trauma cases [52,53].

It should be emphasized that determining whether seizure prophylaxis succeeds in preventing postoperative seizures in patients with brain tumors is challenging due to the intricate pathologic processes and the existence of several predisposing factors, such as the tumor size and type, the craniotomy location, as well as the extent of resection [15].

Based on a systematic literature review, the majority of documented adverse effects associated with AED prophylaxis range from 15% to 24% and are not considered serious [14]. The implementation of novel antiepileptic medications like levetiracetam can reduce this risk even further. A recently published meta-analysis [13] registered an augmented risk of adverse effects in phenytoin-treated patients compared to those receiving levetiracetam (15.5% versus 7.5%, respectively). Over and above, levetiracetam has been reported to enhance the sensitivity of glioblastoma to chemotherapy, with some investigators suggesting that levetiracetam should be used as a first-line treatment for individuals suffering from brain tumors [13,47].

Levetiracetam is a pyrridoline derivative with greater potency and an attractive pharmacokinetic profile. It has limited plasma protein binding, great bioavailability, linear kinetics, a rapid rate of reaching steady-state concentrations, and a comparatively wide therapeutic window that does not require slow titration or serological monitoring at standard doses [47]. There have been fewer drug–drug interactions since levetiracetam is predominantly metabolized in the kidneys, where CYP enzymes are not engaged [13].

Our findings seem to reinforce existing evidence, considering the low ranking of phenytoin in safety outcomes assessment, namely, mortality and adverse effects. On the other hand, levetiracetam demonstrated enhanced safety performance in terms of mortality, ranking second after placebo, and improved tolerability compared to phenytoin, indicating its considerable potential. Discontinuation due to adverse effects was more common with phenytoin than with levetiracetam. Nonetheless, none of the safety-relevant outcomes in our analysis exhibited any statistical significance.

It should be emphasized that the frequency and magnitude of AED-related side effects should be interpreted in light of their potential clinical efficacy. A significant therapeutic or preventive benefit may warrant adverse effects, while a negligible or uncertain benefit does not warrant any degree of risk. A risk-benefit analysis from the included studies would have been largely out of date even if network outcomes for safety were more consistently reported because the included studies used first-generation AEDs (phenytoin, carbamazepine, valproic acid, and phenobarbital) for seizure prophylaxis rather than second-generation AEDs like levetiracetam, which are known to be more tolerable. Determining whether a newer generation AED will be beneficial for a population of seizure-naïve patients, given the possible risk reduction, is the next step towards definitively ending this lengthy but significant controversy. The estimation of the benefits of AEDs may vary if side effects occur less frequently or if a specific high-risk category is targeted [13].

It should be emphasized that rankings for early seizures and major AEs are descriptive, given the absence of closed loops; mortality comparisons showed no significant differences with sparse data.

Several limitations should be acknowledged and addressed in this network meta-analysis. First, the sample sizes for our pooled analyses are fairly limited since there are very few RCTs of AED prophylaxis with extractable data from patients undergoing craniotomy for various brain pathologies. Second, the implementation of seizure prophylaxis differed in timing, routes or dosing, duration of treatment, and the administration of AEDs either as monotherapy or as a mixture of anticonvulsants. Third, there was a notable variability in the time frame of outcomes assessment, with the interventions relevant to short-term analysis being more consistent compared to those included in the analysis of long-term outcomes. Fourth, the included studies are heterogeneous in terms of the type and location of the primary brain tumor, the complexity of the surgical intervention, as well as the incorporation of either seizure-naive individuals or individuals who experience seizures. Notably, only five RCTs included exclusively brain tumor populations, with the remaining incorporating other surgically treated brain pathologies. Lastly, the uncertainty in the risk of bias in the included trials, as well as the moderate to low-quality network analysis of study endpoints, might have attenuated the validity of the reported finding.

## 5. Conclusions

The findings of our network meta-analysis indicate that, in individuals without a history of seizures undergoing craniotomy for brain tumor excision, levetiracetam effectively prevents postoperative total and short-term seizure activity and emerges as a more advantageous anticonvulsant drug than phenytoin and valproate, while phenytoin seems to be superior only to placebo. Nonetheless, none of the tested AEDs modulated late seizure development. Moreover, levetiracetam presents an enhanced safety profile in terms of unfavorable outcomes, yet no statistical superiority over other AEDs could be demonstrated. Nonetheless, the limited current evidence precludes any definite recommendation based on the routine use of AEDs in clinical practice for post-craniotomy seizure prophylaxis.

Thus, there is an urgent need for more well-designed, high-quality, and head-to-head comparisons with other control group trials to comprehensively evaluate the effectiveness of AEDs for seizure prevention following cranial surgery and to generate enough data to identify the most appropriate AED if prophylactic treatment is needed. The top priorities of future trials should be to overcome the methodological inconsistencies encountered in this analysis and to define the timing of AED administration (pre- or post-surgery), as well as the adequate length of treatment or follow-up period.

## Figures and Tables

**Table 1:** League table demonstrating the relative effectiveness of each pair of comparisons for total seizures. Odds ratios greater than 1 favor interventions in the column; forest plots use the conventional orientation (values < 1 favor the intervention versus placebo). Treatment modalities and statistically significant findings are highlighted using bold font.

**CBZ**							
0.28 (0.01; 8.27)	**GAB**						
0.16 (0.03; 0.91)	0.58 (0.02; 20.58)	**LEV**					
1.02 (0.43; 2.39)	3.57 (0.12; 103.11)	**6.20 (1.13; 34.09)**	**No treatment**				
0.85 (0.36; 2.04)	3.00 (0.12; 77.79)	**5.21 (1.19; 22.91)**	0.84 (0.36; 1.95)	**PHT**			
0.37 (0.08; 1.78)	1.31 (0.04; 48.36)	2.28 (0.27; 19.49)	0.37 (0.10; 1.35)	0.44 (0.09; 2.07)	**PHT/PB**		
1.08 (0.39; 3.05)	3.81 (0.14; 103.66)	**6.62 (1.36; 32.21)**	1.07 (0.39; 2.93)	1.27 (0.73; 2.22)	2.91 (0.56; 15.14)	**PBO**	
1.01 (0.30; 3.37)	3.56 (0.12; 102.51)	**6.19 (1.13; 33.77)**	1.00 (0.31; 3.26)	1.19 (0.52; 2.72)	2.72 (0.47; 15.81)	0.93 (0.34; 2.54)	**VAL**

**Table 2:** League table demonstrating the relative effectiveness of each pair of comparisons for early seizure control. Odds ratios greater than 1 favor interventions in the column; forest plots use the conventional orientation (values < 1 favor the intervention versus placebo). Treatment modalities and statistically significant findings are highlighted using bold font.

**GAB**				
0.57 (0.02; 19.93)	**LEV**			
3.00 (0.12; 76.03)	**5.23 (1.21; 22.57)**	**PHT**		
6.93 (0.25; 191.33)	**12.08 (2.34; 62.43)**	**2.31 (1.09; 4.88)**	**PBO**	
4.72 (0.17; 133.01)	**8.23 (1.53; 44.29)**	1.57 (0.68; 3.62)	0.68 (0.22; 2.09)	**VAL**

**Table 3:** League table demonstrating the relative effectiveness of each pair of comparisons for late seizures. Odds ratios greater than 1 favor interventions in the column; forest plots use the conventional orientation (values < 1 favor the intervention versus placebo). Treatment modalities are highlighted using bold font.

**CBZ**						
1.02 (0.07; 14.99)	**No treatment**					
1.89 (0.04; 92.78)	1.85 (0.08; 43.16)	**PB**				
2.80 (0.19; 42.00)	2.75 (0.29; 25.85)	1.48 (0.05; 40.24)	**PHT**			
1.14 (0.03; 41.19)	1.12 (0.04; 28.84)	0.60 (0.01; 34.78)	0.41 (0.04; 4.28)	**PBO**		
1.61 (0.03; 98.49)	1.58 (0.03; 72.28)	0.85 (0.01; 78.78)	0.57 (0.03; 12.73)	1.41 (0.03; 68.75)	**VAL**	
0.80 (0.01; 112.65)	0.79 (0.01; 62.92)	0.43 (0.02; 8.94)	0.29 (0.00; 25.64)	0.70 (0.00; 111.9)	0.50 (0.00; 117.12)	**ZNS**

## Data Availability

The original contributions presented in this study are included in the article/Supplementary Material. Further inquiries can be directed to the corresponding author.

## Supplementary Materials

The following supporting information can be downloaded at https://www.mdpi.com/article/10.3390/jcm14217854/s1. Table S1: PRISMA Checklist; Table S2: PUBMED Search strategy; Table S3: CENTRAL Search strategy; Figure S1: Web of Science Search strategy.

## Abbreviations

The following abbreviations are used in this manuscript:

RCT	Randomized Controlled Trial
AED	Anticonvulsant Drug
NMA	Network Meta Analysis
CINeMA	Confidence in Network Meta-Analysis
CI	Confidence Interval
OR	Odds Ratio

## Appendix A

**Table A1:** Characteristics of the included studies.

Study ID	Study Design	No of pts	Study Arms	Brain Pathology	Treatment Duration	Follow-Up	
						Early	Late
Beenen et al., 1999 [32]	Double-blind RCT	100	PHT 300 mg/d (iv) vs. VAL 1500 mg/d (iv) post-op	Vascular lesions, tumor, trauma	12 mo	1 wk	2 wks–12 mo
Zhang et al., 2000 [33]	RCT	152	PHT 10 mg/kg × 3 daily (oral) for 7 d pre-op & 5 mg/kg × 3 daily (iv) for 2 d & 5 mg/kg × 3 daily (oral) for 1 mo post-op vs. VAL 30 mg/kg × 3 daily (oral) for 7 d pre-op & 20 mg/kg × 3 daily (iv) for 2 d & 20 mg/kg × 2 daily (oral) for 1 mo post-op	Vascular lesions, tumor, trauma	1 mo	1 wk	>3 mo
Foy et al., 1992 [34]	Open-label, controlled RCT	276	PHT (15 mg/kg for 24 h pre-op & 300 mg/d post-op) vs. CBZ (800 mg for 24 h pre-op & 600 mg/d post-op) vs. NT	Vascular lesions, abscess, tumor	6 & 24 mo	NR	4 yrs (median)
Fuller et al., 2013 [35]	Single-blind RCT	81	PHT 300 mg × 3/d to 1 gr/d (iv) preop & 300 mg (oral) postop vs. LEV 500 mg–1 g/d (iv or oral) Preop (3 d) & post-op (3 mo)	Vascular lesions tumor, hematoma, abscess	3 mo	3 d & hospital discharge	3 mo
Iuchi et al., 2015 [36]	Open-cohort RCT	146	PHT 15–18 mg/kg & 5–7.5 mg/kg/d iv & 250 mg/d (oral) vs. LEV 500 mg iv & 1 g/d (sup or oral) intra-op & post-op	Tumors	7 d	1 wk	NA
Faghihjouibari et al., 2023 [37]	Double-blind, RCT	80	PHT 300 mg/d (iv or oral) vs. LEV 1 g/d (oral) preop (5 d) & post-op	Tumor	3 mo	1 wk	1 mo & 3 mo
Türe et al., 2009 [38]	RCT	80	PHT 300 mg/d (oral) vs. GAB 1200 mg/d (oral) 7 d pre-op	Tumor	6–12 mo	1 wk	1 mo
Lee et al., 1989 [39]	RCT	374	PHT (15 mg/kg end surgery & 5–6 mg/kg/d for 3 d post-op iv) vs. PBO	Vascular lesions tumor, hematoma, trauma	3 d	3 d	NA
North et al., 1983 [40]	Double-blind, RCT	281	PHT 500 mg/d (iv) & 300 mg/d (oral) post-op vs. PBO	Vascular lesions, tumor, trauma	12 mo	1 wk	12 mo
Wu et al., 2013 [41]	RCT	123	PHT 15 mg/kg (iv) pre-op & 300 mg/d (iv or peros) post-op for 7 d vs. PBO	Tumor	7 d	1 wk	>30 d
Franceschetti et al., 1990 [42]	RCT	63	PHT 10 mg/kg (iv for 5 d) & then 5 mg/kg (oral) vs. PB 4 mg/kg (iv for 5 d) & then 2 mg/kg (oral) vs. NT	Tumor	Unclear	1 wk	Unclear
Nakamura et al., 1999 [43]	Double-blind RCT	255	ZNS 200 mg/d (iv) vs. PB 80 mg/d (oral) for 1 mo pre-op & post-op	Vascular lesions, tumor, trauma	12 mo	NA	1–12 mo

ABBREVIATIONS. RCT, randomized controlled trial; PHT, phenytoin; VAL, valproic acid; CBZ, carbamazepine; LEV, levetiracetam; PB, phenobarbital; GAB, gabapentin; ZNS, zonisamide; NT, no-treatment; PBO, placebo; mo, month; wk, week; d, days; h, hours; iv, intravenous; pre-op, preoperatively; post-op, postoperatively; pts, patients; NA, not assessed.

**Table A2:** Outcome parameters of the included studies.

Study ID	Primary Outcome				Secondary Outcomes		
	All Seizures		Early Seizures	Late Seizures	Mortality	Discontinuation Due to AEs	Other Adverse Events
	NT or PBO	Control AED					
Beenen et al., 1999 [32]	-	PHT (7/50, 14%) vs. VAL (7/50, 14%) [NS]	PHT (4/50, 8%) vs. VAL (2/50, 4%) [NS]	PHT (3/50, 6%) vs. VAL (5/50, 10%) [NS]	PHT (13/50%, 26%) vs. VAL (10/50, 20%)	Skin rashes, PONV in PHT (5/50, 10%) vs. liver dysfunction, thrombopenia in VAL (2/50, 4%)	Anemia, PONV in PHT (4/50, 8%) vs. weight gain, PONV in VAL (2/50, 4%) [NS]
Zhang et al., 2000 [33]	-	PHT (6/72, 8%) vs. VAL (9/80, 11%)	PHT (6/72, 8%) vs. VAL (9/80, 11%)	NA	NA	NA	PHT (11/52, 15%) vs. VAL (2/80, 3%) [*p* < 0.05]
Foy et al., 1992 [34]	NT (25/59, 42%) 6-mo NT (20/59, 42%) 24 mo	PHT (21/55, 38%) vs. CBZ (21/50, 42%) 6-mo PHT (16/56, 29%) vs. CBZ (20/56, 36%) 24 mo	NA	PHT (21/55, 38%) vs. CBZ (21/50, 42%) vs. NT (25/59, 42%) 6-mo PHT (16/56, 29%) vs. CBZ (20/56, 36%) vs. NT (20/59, 42%) 24 mo	CBZ (19/106) vs. PHT (32/111) & vs. NT (13/59) [NS]	Skin rashes (28/217, 13%) Drug intoxication (6/217, 3%)	
Fuller et al., 2013 [35]	-	PHT (6/42, 14%) vs. LEV (0/30, 0%) up to 6 d [*p* = 0.01]	PHT (6/42, 14%) vs. LEV (0/30, 0%) up to 6 d [*p* = 0.01]	NA	PHT (5/42, 12%) vs. LEV (3/39, 8%)	Allergic reactions PHE iv (2/38, 5%) vs. skin rash LEV iv (1/36, 3%) Intoxication, rash, ataxia, nausea PHE oral (3/38, 8%) vs. delirium, pruritus, & headache LEV oral (3/36, 8%) [NS]	Thrombophlebitis PHT (3/38, 8%) Mood disturbance PHT (3/38, 8%) vs. LEV (7/36, 19%) PHT AEs (18/42, 43%) vs. LEV (22/39, 56%)
Iuchi et al., 2015 [36]	-	PHT (8/53, 14%) vs. LEV (1/53, 1.9%) [*p* = 0.034]	PHT (8/53, 14%) vs. LEV (1/53, 1.9%) [*p* = 0.034]	NA	NA	PHT 5/73, 6.8%) due to liver dysfunction, skin eruption & AF vs. LEV 0 [*p* = 0.058]	PHT (8/73, 11%) vs. LEV (3/73, 4%) [NS]
Faghihjouibari et al., 2023 [37]	-	PHT (1/40, 2.5%) vs. LEV (1/39, 2.6%) [NS]	PHE (1/40, 2.5%) 6 d vs. LEV (1/39, 2.6%) intra-op	None in either group	NA	None in either group	Skin rash PHT (3/40, 7.5%) vs. LEV 0% [NS] Thrombocytopenia PHT (1/40, 2.5%) vs. LEV 0%
Türe et al., 2009 [38]	-	PHT (1/38, 3%) vs. GAB 0%	PHT (1/38, 3%) vs. GAB 0% [NS]	PHE 0% vs. GAB 0%	PHT 0% vs. GAB 0%	Fatigue & dizziness GAB (2/37, 5%) vs. PHT (0/37, 0%)	Fatigue & dizziness GAB (10/37, 27%) vs. PHT 0% Ataxia, myalgia, PONV in GAB (12/37, 32%) vs. ataxia, PONV in PHT (18/38, 47%)
Lee et al., 1989 [39]	PHT (2/189, 1%) vs. PBO (9/285, 5%)	-	PHT (2/189, 1%) vs. PBO (9/285, 5%)	NA	NA	NA	NA
North et al., 1983 [40]	PHT (18/140, 13%) vs. PBO (26/141, 18%)	-	PHT (4/140, 3%) vs. PBO (14/141, 20%) [*p* < 0.05]	PHT (14/140, 10%) vs. PBO (12/141, 8.5%)	PHT (20/140, 14%) vs. PBO (12/140, 9%) [NS]	Rashes (8/140, 6%) Involuntary movements, hirsutism, headache, discomfort of the face (1 pt/effect) in PHT vs. Rash, dizziness & nausea (1 pt/effect) in PBO	
Wu et al., 2013 [41]	PHT (15/62, 24%) vs. PBO (11/61, 18%)	-	PHT (5/62, 8%) vs. PBO (5/61, 8%) [NS]	PHT (9/62, 14%) vs. PBO (6/61, 10%) [NS]	NA	Major AEs PHT (3/62, 5%) vs. PBO (0/61, 0%)	Minor AEs PHT (9/62, 15%) vs. PBO 0% [*p* < 0.01]
Franceschetti et al., 1990 [42]	PHT or PB (6/41, 15%) vs. NT (7/22, 32%)		Total PHT or PB (3/41, 7%) vs. NT (4/22, 18%)	PHT (1/10, 10%) vs. PB (2/15, 13%) vs. NT (3/14, 21%) [NS]	NA	NA	Neurological AEs PHT (3/16, 19%) vs. PB (1/25, 4%) within 7 d
Nakamura et al., 1999 [43]	-	ZNS (13/129, 10%) vs. PB (11/126, 9%)	ZNS (6/129, 5%) vs. PB (3/126, 2%)	ZNS (7/129, 5%) vs. PB (8/126, 6%)	ZNS (8/112, 7%) vs. PB (13/107, 12%)	NA	ZNS (28/129, 22%) vs. PB (30/126, 24%)

ABBREVIATIONS. RCT, randomized controlled trial; GAB, gabapentin; PHT, phenytoin; VAL, valproic acid; CBZ, carbamazepine; LEV, levetiracetam; PB, phenobarbital; ZNS, zonisamide; NT, no-treatment; PBO, placebo; PONV, postoperative nausea and vomiting; AEs, adverse events; mo, month; wk, week; d, days; h, hours; iv, intravenous; pre-op, preoperatively; post-op, postoperatively; pts, patients; NA, not assessed; NS, non-significant.

**Table A3:** League table demonstrating the relative effectiveness of each pair of comparisons for mortality. Odds ratios greater than 1 favor interventions in the column; forest plots use the conventional orientation (values < 1 favor the intervention versus placebo). Treatment modalities are highlighted using bold font.

**CBZ**					
3.01 (0.59; 15.43)	**LEV**	.			
1.43 (0.68; 3.00)	0.48 (0.09; 2.60)	**No treatment**			
1.85 (0.97; 3.53)	0.62 (0.14; 2.77)	1.29 (0.59; 2.85)	**PHT**		
3.30 (1.22; 8.91)	1.10 (0.20; 5.90)	2.30 (0.77; 6.88)	1.78 (0.83; 3.79)	**PBO**	
2.61 (0.84; 8.13)	0.87 (0.15; 5.10)	1.82 (0.53; 6.20)	1.41 (0.55; 3.59)	0.79 (0.24; 2.64)	**VAL**

**Table A4:** League table demonstrating the relative contribution of each pair of comparisons to major adverse effects. Odds ratios greater than 1 favor interventions in the column; forest plots use the conventional orientation (values < 1 favor the intervention versus placebo). Treatment modalities are highlighted using bold font.

**GAB**				
10.08 (0.36; 279.10)	**LEV**			
5.42 (0.25; 116.90)	0.54 (0.15; 1.90)	**PHT**		
25.36 (0.94; 681.34)	2.52 (0.45; 14.20)	4.68 (1.43; 15.27)	**PBO**	
14.46 (0.43; 481.28)	1.43 (0.17; 11.83)	2.67 (0.49; 14.45)	0.57 (0.07; 4.49)	**VAL**

**Table A5:** CINeMA summary tables for primary outcomes. CINeMA components are highlighted using bold font.

Comparison	No	Within-Study Bias	Reporting Bias	Indirectness	Imprecision	Heterogeneity	Incoherence	Confidence Rating
Carbamazepime: No treatment	1	Major concerns	Some concerns	Some concerns	Some concerns	Some concerns	Major concerns	Moderate
Carbamazepime: Phenyntoin	1	Major concerns	Some concerns	Some concerns	Some concerns	Some concerns	Major concerns	Moderate
Gabapentin: Phenyntoin	1	Some concerns	Some concerns	No concerns	Major concerns	No concerns	Major concerns	Low
Levetiracetam: Phenyntoin	3	Some concerns	Some concerns	Some concerns	No concerns	Major concerns	Major concerns	Low
No treatment: Phenyntoin	1	Major concerns	Some concerns	Some concerns	Some concerns	Some concerns	Major concerns	Low
No treatment: Phenyntoin/Phenobarbital	1	Major concerns	Some concerns	Major concerns	Major concerns	Some concerns	Major concerns	Very low
Phenyntoin: Placebo	3	Some concerns	Some concerns	No concerns	Some concerns	Some concerns	Major concerns	Moderate
Phenyntoin: Valproate	2	Some concerns	Some concerns	Some concerns	Major concerns	Some concerns	Major concerns	Moderate
Carbamazepime: Gabapentin	0	Major concerns	Some concerns	Some concerns	Major concerns	Major concerns	Major concerns	Very low
Carbamazepime: Levetiracetam	0	Major concerns	Some concerns	Some concerns	Major concerns	Major concerns	Major concerns	Very low
Carbamazepime: Phenyntoin/Phenobarbital	0	Major concerns	Some concerns	Some concerns	Major concerns	No concerns	Major concerns	Low
Carbamazepime: Placebo	0	Major concerns	Some concerns	Some concerns	Major concerns	Some concerns	Major concerns	Very low
Carbamazepime: Valproate	0	Major concerns	Some concerns	Some concerns	Major concerns	Some concerns	Major concerns	Very low
Gabapentin: Levetiracetam	0	Some concerns	Some concerns	No concerns	Major concerns	No concerns	Major concerns	Moderate
Gabapentin: No treatment	0	Major concerns	Some concerns	Some concerns	Major concerns	No concerns	Major concerns	Low
Gabapentin: Phenyntoin/Phenobarbital	0	Major concerns	Some concerns	Some concerns	Major concerns	No concerns	Major concerns	Low
Gabapentin: Placebo	0	Some concerns	Some concerns	No concerns	Major concerns	No concerns	Major concerns	Low
Gabapentin: Valporate	0	Some concerns	Some concerns	No concerns	Major concerns	No concerns	Major concerns	Very low
Levetiracetam: No treatment	0	Major concerns	Some concerns	Some concerns	No concerns	Major concerns	Major concerns	Very low
Levetiracetam: Phenyntoin/Phenobarbital	0	Major concerns	Some concerns	Some concerns	Major concerns	No concerns	Major concerns	Low
Levetiracetam: Placebo	0	Major concerns	Some concerns	No concerns	No concerns	No concerns	Major concerns	Moderate
Levetiracetam: Valproate	0	Some concerns	Some concerns	Some concerns	No concerns	No concerns	Major concerns	Low
No treatment: Placebo	0	Major concerns	Some concerns	Some concerns	Major concerns	Major concerns	Major concerns	Very low
No treatment: Valproate	0	Major concerns	Some concerns	Some concerns	Major concerns	Major concerns	Major concerns	Very low
Phenyntoin: : Phenyntoin/Phenobarbital	0	Major concerns	Some concerns	Some concerns	Major concerns	No concerns	Major concerns	Low
Phenyntoin/Phenobarbital: Placebo	0	Major concerns	Some concerns	Some concerns	Some concerns	No concerns	Major concerns	Moderate
Phenyntoin/Phenobarbital: Valproate	0	Major concerns	Some concerns	Some concerns	Some concerns	No concerns	Major concerns	Moderate
Placebo: Valproate	0	Some concerns	Some concerns	No concerns	Some concerns	No concerns	Major concerns	Moderate

**Table A6:** CINeMA summary tables for secondary outcomes. CINeMA components are highlighted using bold font.

Comparison	No	Within-Study bias	Reporting Bias	Indirectness	Imprecision	Heterogeneity	Incoherence	Confidence Rating
GAB:PHT	1	Some concerns	Some concerns	No concerns	Major concerns	No concerns	Major concerns	Low
LEV:PHT	3	Major concerns	Some concerns	Some concerns	Major concerns	No concerns	Major concerns	Very low
NT:PHE	1	Major concerns	Some concerns	Some concerns	Major concerns	No concerns	Major concerns	Very low
NT:PHT	1	Major concerns	Some concerns	Some concerns	Major concerns	No concerns	Major concerns	Very low
PBO:PHT	2	Some concerns	Some concerns	No concerns	No concerns	Major concerns	Major concerns	Very low
PHE:PHT	1	Major concerns	Some concerns	Some concerns	Major concerns	No concerns	Major concerns	Very low
PHT:VAL	2	Some concerns	Some concerns	No concerns	Major concerns	No concerns	Major concerns	Low
GAB:LEV	0	Major concerns	Some concerns	Some concerns	Major concerns	No concerns	Major concerns	Very low
GAB:NT	0	Major concerns	Some concerns	Some concerns	Major concerns	No concerns	Major concerns	Very low
GAB:PBO	0	Some concerns	Some concerns	No concerns	Major concerns	No concerns	Major concerns	Very low
GAB:PHE	0	Major concerns	Some concerns	Some concerns	Major concerns	No concerns	Major concerns	Very low
GAB:VAL	0	Some concerns	Some concerns	No concerns	Major concerns	No concerns	Major concerns	Low
LEV:NT	0	Major concerns	Some concerns	Some concerns	Major concerns	No concerns	Major concerns	Very low
LEV:PBO	0	Major concerns	Some concerns	Some concerns	Major concerns	No concerns	Major concerns	Very low
LEV:PHE	0	Major concerns	Some concerns	Some concerns	Major concerns	No concerns	Major concerns	Very low
LEV:VAL	0	Major concerns	Some concerns	Some concerns	Major concerns	No concerns	Major concerns	Very low
NT:PBO	0	Major concerns	Some concerns	Some concerns	Major concerns	No concerns	Major concerns	Very low
NT:VAL	0	Major concerns	Some concerns	Some concerns	Major concerns	No concerns	Major concerns	Very low
PBO:PHE	0	Major concerns	Some concerns	Some concerns	Major concerns	No concerns	Major concerns	Very low
PBO:VAL	0	Some concerns	Some concerns	No concerns	Major concerns	No concerns	Major concerns	Low
PHE:VAL	0	Major concerns	Some concerns	Some concerns	Major concerns	No concerns	Major concerns	Very low
