# Supplementary material for: Antiepileptic Drugs for De Novo Seizure Prevention After Craniotomy: A Systematic Review and Network Meta-Analysis of Current Evidence
Source: J Clin Med. 2025 Nov 5;14(21):7854. doi: 10.3390/jcm14217854 (PMC12608386; doi:10.3390/jcm14217854)
Supplement: Supplementary file 1 [file jcm-14-07854-s001.zip › Supplementary File Table S2, S3 Figure S1.pdf]

Supplementary data

Table S2: PUBMED Search strategy

Search number	Query	Search Details
1	(brain tumor) AND ((seizure)) OR (epilepsy))	(("brain tumour"[All Fields] OR "brain neoplasms"[MeSH Terms] OR ("brain"[All Fields] AND "neoplasms"[All Fields]) OR "brain neoplasms"[All Fields] OR ("brain"[All Fields] AND "tumor"[All Fields]) OR "brain tumor"[All Fields]) AND ("seizural"[All Fields] OR "seizure s"[All Fields] OR "seizured"[All Fields] OR "seizures"[MeSH Terms] OR "seizures"[All Fields] OR "seizure"[All Fields] OR "seizuring"[All Fields])) OR ("epilepsie"[All Fields] OR "epilepsy"[MeSH Terms] OR "epilepsy"[All Fields] OR "epilepsies"[All Fields] OR "epilepsy s"[All Fields])
2	(antiepileptic) OR (anticonvulsant)	"anticonvulsants"[Pharmacological Action] OR "anticonvulsants"[MeSH Terms] OR "anticonvulsants"[All Fields] OR "antiepileptic"[All Fields] OR "antiepileptics"[All Fields] OR "anticonvulsants"[Pharmacological Action] OR "anticonvulsants"[MeSH Terms] OR "anticonvulsants"[All Fields] OR "anticonvulsant"[All Fields] OR "anticonvulsion"[All Fields] OR "anticonvulsive"[All Fields] OR "anticonvulsives"[All Fields]
3	(#1) AND (#2)	((("brain tumour"[All Fields] OR "brain neoplasms"[MeSH Terms] OR ("brain"[All Fields] AND "neoplasms"[All Fields]) OR "brain neoplasms"[All Fields] OR ("brain"[All Fields] AND "tumor"[All Fields]) OR "brain tumor"[All Fields]) AND ("seizural"[All Fields] OR "seizure s"[All Fields] OR "seizured"[All Fields] OR "seizures"[MeSH Terms] OR "seizures"[All Fields] OR "seizure"[All Fields] OR "seizuring"[All Fields])) OR ("epilepsie"[All Fields] OR "epilepsy"[MeSH Terms] OR "epilepsy"[All Fields] OR "epilepsies"[All Fields] OR "epilepsy s"[All Fields])) AND ("anticonvulsants"[Pharmacological Action] OR "anticonvulsants"[MeSH Terms] OR "anticonvulsants"[All Fields] OR "antiepileptic"[All Fields] OR "antiepileptics"[All Fields] OR ("anticonvulsants"[Pharmacological Action] OR "anticonvulsants"[MeSH Terms] OR "anticonvulsants"[All Fields] OR "anticonvulsant"[All Fields] OR "anticonvulsion"[All Fields] OR "anticonvulsive"[All Fields] OR "anticonvulsives"[All Fields]))
4	(#3) AND (prophyla*)	((("brain tumour"[All Fields] OR "brain neoplasms"[MeSH Terms] OR ("brain"[All Fields] AND "neoplasms"[All Fields]) OR "brain neoplasms"[All Fields] OR ("brain"[All Fields] AND "tumor"[All Fields]) OR "brain tumor"[All Fields]) AND ("seizural"[All Fields] OR "seizure s"[All Fields] OR "seizured"[All Fields] OR "seizures"[MeSH Terms] OR "seizures"[All Fields] OR "seizure"[All Fields] OR "seizuring"[All Fields])) OR ("epilepsie"[All Fields] OR "epilepsy"[MeSH Terms] OR "epilepsy"[All Fields] OR "epilepsies"[All Fields] OR "epilepsy s"[All Fields])) AND ("anticonvulsants"[Pharmacological Action] OR "anticonvulsants"[MeSH Terms] OR "anticonvulsants"[All Fields] OR "antiepileptic"[All Fields] OR "antiepileptics"[All Fields] OR ("anticonvulsants"[Pharmacological Action] OR "anticonvulsants"[MeSH Terms] OR "anticonvulsants"[All Fields] OR "anticonvulsant"[All Fields] OR "anticonvulsion"[All Fields] OR "anticonvulsive"[All Fields] OR "anticonvulsives"[All Fields])) AND "prophyla*"[All Fields]
5	craniotomy	"craniotomy"[MeSH Terms] OR "craniotomy"[All Fields] OR "craniotomies"[All Fields]
6	(#4) AND (#5)	((("brain tumour"[All Fields] OR "brain neoplasms"[MeSH Terms] OR ("brain"[All Fields] AND "neoplasms"[All Fields]) OR "brain neoplasms"[All Fields] OR ("brain"[All Fields] AND "tumor"[All Fields]) OR "brain tumor"[All Fields]) AND ("seizural"[All Fields] OR "seizure s"[All Fields] OR "seizured"[All Fields] OR "seizures"[MeSH Terms] OR "seizures"[All Fields] OR "seizure"[All Fields] OR "seizuring"[All Fields])) OR ("epilepsie"[All Fields] OR "epilepsy"[MeSH Terms] OR "epilepsy"[All Fields] OR "epilepsies"[All Fields] OR "epilepsy s"[All Fields])) AND ("anticonvulsants"[Pharmacological Action] OR "anticonvulsants"[MeSH Terms] OR "anticonvulsants"[All Fields] OR "antiepileptic"[All Fields] OR "antiepileptics"[All Fields] OR ("anticonvulsants"[Pharmacological Action] OR "anticonvulsants"[MeSH Terms] OR "anticonvulsants"[All Fields] OR "anticonvulsant"[All Fields] OR "anticonvulsion"[All Fields] OR "anticonvulsive"[All Fields] OR "anticonvulsives"[All Fields])) AND "prophyla*"[All Fields] AND ("craniotomy"[MeSH Terms] OR "craniotomy"[All Fields] OR "craniotomies"[All Fields])
7	(side effects) OR (adverse effects)	"adverse effects"[MeSH Subheading] OR ("adverse"[All Fields] AND "effects"[All Fields]) OR "adverse effects"[All Fields] OR ("side"[All Fields] AND "effects"[All Fields]) OR "side effects"[All Fields] OR ("adverse effects"[MeSH Subheading] OR ("adverse"[All Fields] AND "effects"[All Fields]) OR "adverse effects"[All Fields])
8	((#3) AND (#5)) AND (#7)	((("brain tumour"[All Fields] OR "brain neoplasms"[MeSH Terms] OR ("brain"[All Fields] AND "neoplasms"[All Fields]) OR "brain neoplasms"[All Fields] OR ("brain"[All Fields] AND "tumor"[All Fields]) OR "brain tumor"[All Fields]) AND ("seizural"[All Fields] OR "seizure s"[All

		Fields] OR "seizured"[All Fields] OR "seizures"[MeSH Terms] OR "seizures"[All Fields] OR "seizure"[All Fields] OR "seizuring"[All Fields])) OR ("epilepsie"[All Fields] OR "epilepsy"[MeSH Terms] OR "epilepsy"[All Fields] OR "epilepsies"[All Fields] OR "epilepsy s"[All Fields])) AND ("anticonvulsants"[Pharmacological Action] OR "anticonvulsants"[MeSH Terms] OR "anticonvulsants"[All Fields] OR "antiepileptic"[All Fields] OR "antiepileptics"[All Fields] OR ("anticonvulsants"[Pharmacological Action] OR "anticonvulsants"[MeSH Terms] OR "anticonvulsants"[All Fields] OR "anticonvulsant"[All Fields] OR "anticonvulsion"[All Fields] OR "anticonvulsive"[All Fields] OR "anticonvulsives"[All Fields])) AND ("craniotomy"[MeSH Terms] OR "craniotomy"[All Fields] OR "craniotomies"[All Fields]) AND ("adverse effects"[MeSH Subheading] OR ("adverse"[All Fields] AND "effects"[All Fields]) OR "adverse effects"[All Fields] OR ("side"[All Fields] AND "effects"[All Fields]) OR "side effects"[All Fields] OR ("adverse effects"[MeSH Subheading] OR ("adverse"[All Fields] AND "effects"[All Fields]) OR "adverse effects"[All Fields]))
9	(#3) AND (prophyla*)	(((("brain tumour"[All Fields] OR "brain neoplasms"[MeSH Terms] OR ("brain"[All Fields] AND "neoplasms"[All Fields]) OR "brain neoplasms"[All Fields] OR ("brain"[All Fields] AND "tumor"[All Fields]) OR "brain tumor"[All Fields]) AND ("seizural"[All Fields] OR "seizure s"[All Fields] OR "seizured"[All Fields] OR "seizures"[MeSH Terms] OR "seizures"[All Fields] OR "seizure"[All Fields] OR "seizuring"[All Fields])) OR ("epilepsie"[All Fields] OR "epilepsy"[MeSH Terms] OR "epilepsy"[All Fields] OR "epilepsies"[All Fields] OR "epilepsy s"[All Fields])) AND ("anticonvulsants"[Pharmacological Action] OR "anticonvulsants"[MeSH Terms] OR "anticonvulsants"[All Fields] OR "antiepileptic"[All Fields] OR "antiepileptics"[All Fields] OR ("anticonvulsants"[Pharmacological Action] OR "anticonvulsants"[MeSH Terms] OR "anticonvulsants"[All Fields] OR "anticonvulsant"[All Fields] OR "anticonvulsion"[All Fields] OR "anticonvulsive"[All Fields] OR "anticonvulsives"[All Fields])) AND "prophyla*"[All Fields]) AND (english[Filter])

Table S3: CENTRAL Search strategy

ID	Search	Hits
#1	craniotomy	1694
#2	brain surgery	10190
#3	#1 OR #2	11196
#4	seizure*	10823
#5	epilepsy	9538
#6	#4 OR #5	15190
#7	antiepileptic*	3249
#8	anticonvulsant*	4577
#9	#7 OR #8	6515
#10	#3 AND #6 in Trials	571
#11	#10 AND #9 in Trials	552

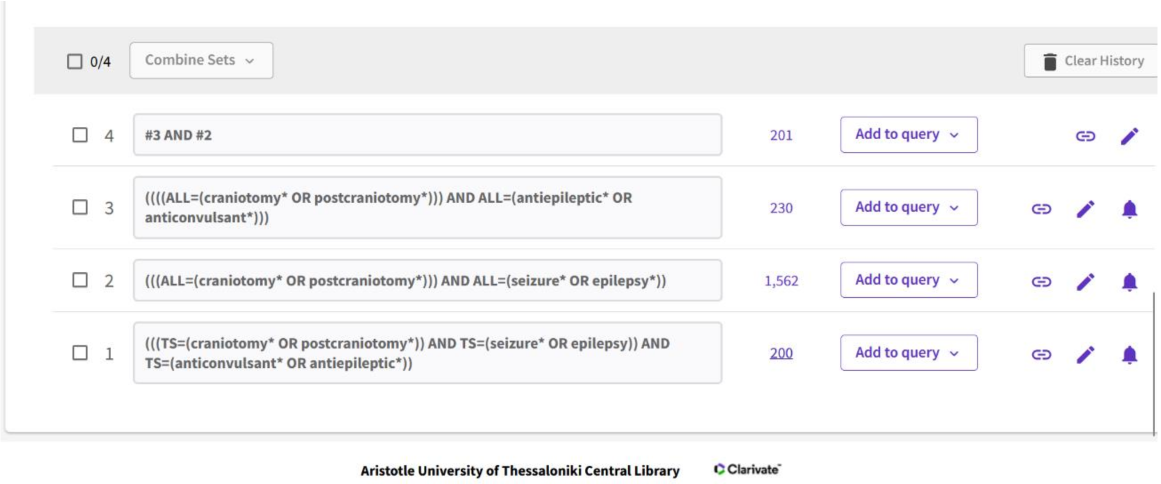

Figure S1: Web of Science Search strategy
